# Ratiometric Electrochemical Sensor Applying SWCNHs/T-PEDOT Nanocomposites for Efficient Quantification of *Tert*-Butylhydroquinone in Foodstuffs

**DOI:** 10.3390/foods13182996

**Published:** 2024-09-21

**Authors:** Jing Wu, Huilin Li, Zhijuan Wang, Mingfei Pan, Shuo Wang

**Affiliations:** 1 Tianjin Key Laboratory of Food Science and Health, School of Medicine, Nankai University, Tianjin 300071, China; wujing2020@nankai.edu.cn; 2 State Key Laboratory of Food Nutrition and Safety, Tianjin University of Science and Technology, Tianjin 300457, China; lhltust@163.com (H.L.); wzhijuan@163.com (Z.W.); panmf2012@tust.edu.cn (M.P.)

**Keywords:** *Tert*-butylhydroquinone, ratiometric electrochemical sensor, SWCNHs, T-PEDOT

## Abstract

*Tert*-butylhydroquinone (TBHQ) is a phenolic substance that is commonly employed to prevent food oxidation. Excessive or improper utilization of this antioxidant can not only impact food quality but may also pose potential risks to human health. In this study, an ultrasensitive, stable, and easily operable ratiometric electrochemical sensor was successfully fabricated by combining the tubular (3,4-ethylenedioxythiophene) (T-PEDOT) with single-wall carbon nanohorns (SWCNHs) for the detection of TBHQ antioxidants in food. The SWCNHs/T-PEDOT nanocomposite fabricated through ultrasound-assisted and template approaches was employed as the modified substrate for the electrode interface. The synergistic effect of SWCNHs and T-PEDOT, which possess excellent electrical conductivity and catalytic properties, enabled the modified electrode to showcase remarkable electrocatalytic performance towards TBHQ, with the redox signal of methylene blue serving as an internal reference. Under optimized conditions, the SWCNHs/T-PEDOT-modified electrode demonstrated good linearity within the TBHQ concentration range of 0.01–200.0 μg mL^−1^, featuring a low limit of detection (LOD) of 0.005 μg mL^−1^. The proposed ratiometric electrochemical sensor displayed favorable reproducibility, stability, and anti-interference capacity, thereby offering a promising strategy for monitoring the levels of TBHQ in oil-rich food products.

## 1. Introduction

*Tert*-butylhydroquinone (TBHQ) is a synthetic phenolic antioxidant that is commonly used to prevent or delay the oxidation of food products. The phenolic hydroxyl group of TBHQ breaks the O-H bond through an electrophilic substitution reaction, releasing hydrogen atoms. The released hydrogen atoms prevent oxidation by combining with free radicals to obtain stabilized products and antioxidant radicals, which interrupt the free radical chain reaction. It usually exists in oil or oil-rich foods, but the illegal addition or excessive use of TBHQ can affect food quality and even pose risks to human health [1,2,3]. To effectively control the potential risk of TBHQ, the maximum allowable residue levels of TBHQ antioxidants in food are strictly regulated in many countries and regions. For instance, both China and the U.S. Food and Drug Administration (FDA) have set the maximum permissible amount of TBHQ in food at 0.2 g kg^−1^, the European Union prohibits the use of TBHQ in soft drinks, while Japan has completely banned its use in food [4,5]. Given these regulations, it is important to develop an accurate, convenient, and sensitive strategy for the detection and monitoring of TBHQ content in food products.

Up to now, a variety of analytical strategies have been developed to detect TBHQ antioxidants in food samples. The chromatographic separation principle and large-scale instruments-based analytical methods such as gas chromatography, liquid chromatography, or combined mass spectrometry have shown advantages of high accuracy, high sensitivity, and good reproducibility [6,7]. However, these technologies involve expensive equipment and complex sample pretreatment processes that are not suitable for on-site and rapid quantitative detection [8,9].

Electrochemical sensors exhibit high sensitivity in rapidly detecting harmful substances in food due to their fast response speeds, low costs, and simplicity of operation, among other characteristics, thus attracting significant attention from researchers, with promising applications [10,11,12]. Nevertheless, the limited conductivity inherent in conventional solid-state working electrodes like glassy carbon affects sensor selectivity and sensitivity, hindering further advancement. To address this issue, various functional materials with distinct structures have been employed to modify electrode surfaces to enhance electrode performance [13,14]. Moreover, traditional electrochemical sensors typically generate a single signal susceptible to external interference from the environment or target states, which may produce ‘false positive’ or ‘false negative’ results. In contrast, ratiometric electrochemical sensors can effectively improve accuracy and reliability by incorporating substances capable of reacting at diverse REDOX potentials while utilizing their signals as references, thereby mitigating interference with targets [15,16]. Single-wall carbon nanohorns (SWCNHs), a novel category of carbon-based nanomaterials, structurally resemble carbon nanotubes and typically aggregate into a “dahlia” form. Owing to their porous architecture, SWCNHs are a promising nanomaterial featuring excellent electrochemical properties, catalytic capacity, and high adsorption capacity [17,18]. Tubular poly (3,4-ethylenedioxythiophene) (T-PEDOT) is a heterocyclic molecular polymer synthesized from the monomer 3, 4-ethylenedioxythiophene, which is a derivative of polythiophene [19]. The conjugated polymer has been demonstrated to possess enhanced electronic stability and improved kinetic parameters [20]. It is well known and frequently utilized as a modified material to enhance the performance of electrochemical sensors [21].

In this study, by combining the tubular (3,4-ethylenedioxythiophene) (T-PEDOT) with single-wall carbon nanohorns (SWCNHs), we have created an ultrasensitive, stable, and easy-to-use ratiometric electrochemical sensor for the detection of TBHQ antioxidants in food. T-PEDOT was synthesized via the template method, and SWCNHs/T-PEDOT nanocomposites were fabricated with the assistance of ultrasound. T-PEDOT not only possessed excellent electrical conductivity and catalytic properties but also had a considerably specific surface area. Its structure offered effective loading for SWCNHs, effectively averting the stacking and aggregation of SWCNHs and facilitating the exposure of active sites. Based on the synergy between SWCNHs/T-PEDOT nanocomposites, a highly catalytic and reliable electrochemical sensing strategy was further developed for the determination of antioxidant TBHQ. This sensing strategy utilized the redox signal of methylene blue as the internal parameter to guarantee the accuracy of target TBHQ detection and provided a convenient and effective approach for the quality control and assurance of oil-rich foods, presenting great application potential.

## 2. Materials and Methods

### 2.1. Chemicals and Materials

The analytes (TBHQ, 98%), epigallocatechin gallate (EGCG, 98%), butylated hydroxytoluene (BHT, 98%), glucose (Glu), hydroquinone (HQ, 98%), propyl gallate (PG, 98%), α-Vitamin E (VE, 98%), ascorbic acid (AA, 98%), methylene blue (MB, 99%) and tri(Hydroxymethyl) amino methane hydrochloride (Tri-HCl, 99%) were purchased from Aladdin Chemical Reagent Co., Ltd. (Shanghai, China). SWCNHs (97%) were acquired from Aiwan Chemical Technology Co., Ltd. (Shanghai, China). The 3,4-ethylenedioxythiophene (EDOT, 98%), ferric chloride (FeCl3, 98%), KCl (99%), NaCl (99%), Na2SO4 (99%), and CH3COONa (99%) were purchased from Shanghai BiDe Pharmaceutical Technology Co., Ltd. (Shanghai, China). Bis(2-ethylhexyl) sulfosuccinate (AOT) and nafion (NF, 5 wt%) were obtained from Sigma-Aldrich (St. Louis, MO, USA). All the reagents utilized in the experiment were of analytical grade.

### 2.2. Instruments

The morphology and structure of the materials synthesized in this work were characterized by field emission scanning electron microscope (SEM, FEI-FEG25, FEI, Hillsboro, OR, USA) and transmission electron microscope (TEM, FEI-F20, FEI, Hillsboro, OR, USA), X-ray powder diffractometer (XRD, SmartLab 9kW, SHIMADZU, Kyoto, Japan), and X-ray photoelectron spectrometer (XPS, ESCALAB Xi+, Thermo, Waltham, MA, USA). The molecular structures of T-PEDOT, SWCNHs, and SWCNHs/T-PEDOT were analyzed using an inVia Reflex spectrometer from Renishaw, UK. Electrochemical experiments were conducted on the CHI440C and Princeton 2273 electrochemical workstation with a three-electrode system.

### 2.3. Synthesis of the SWCNHs/T-PEDOT Composites

**Figure 1:**
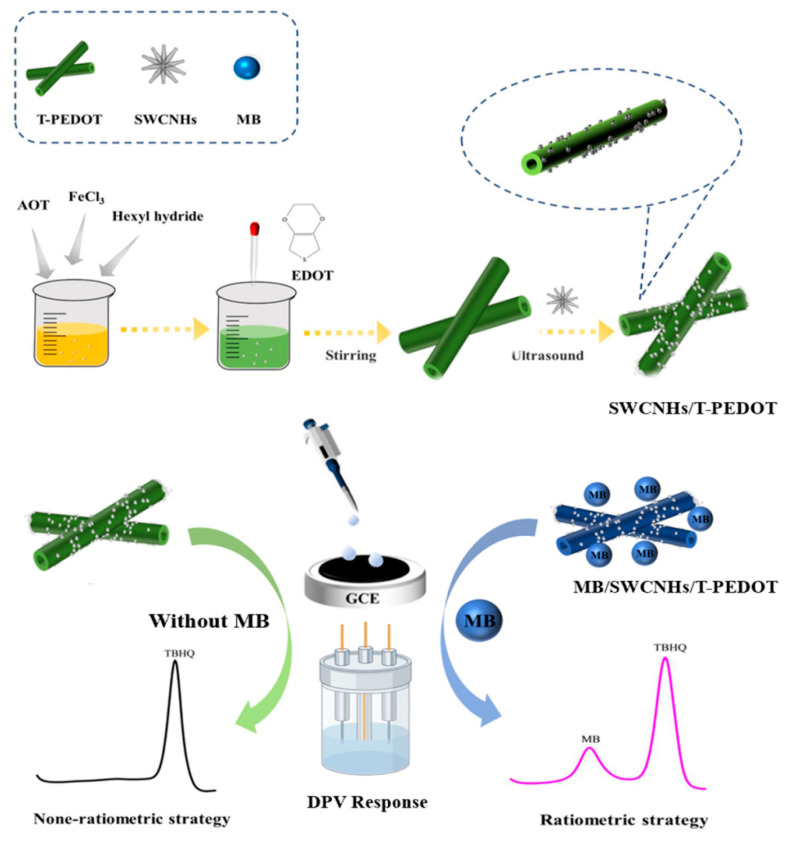
Illustration of the synthesis of SWCNHs/T-PEDOT and the preparation of MB@SWCNHs/T-PEDOT/GCE sensors for TBHQ detection.

The synthesis of T-PEDOT was carried out according to a previously reported method [22,23] with minor modifications (Figure 1). Initially, 8.0 g of sodium AOT was accurately weighed and dissolved in 70.0 mL of hexane with stirring (Solution A). Simultaneously, 1.6 g of FeCl_3_ was accurately weighed and dissolved in 1.0 mL of ultrapure water to prepare Solution B. Subsequently, Solution B was gradually added to Solution A and stirred for 10 min at room temperature, resulting in an orange–yellow solution. While stirring continuously, 0.5 mL of EDOT was added, resulting in a color change from orange–yellow to dark green. Following an additional 3 h of stirring at room temperature, the solution was centrifuged and the resulting solid product (T-PEDOT) was collected and thoroughly washed with acetone and methanol. Finally, the solid product was dried under vacuum at 75 °C for 12 h. The SWCNHs/T-PEDOT nanocomposites were synthesized via ultrasonic replication. Specifically, 2.0 mg of SWCNHs and 2.0 mg of T-PEDOT were individually weighed and dispersed into 2.0 mL of N, N-Dimethylformamide (DMF). The mixture was then ultrasonicated at room temperature for 30 min to obtain a homogeneous dispersion. The two dispersions were then combined and subjected to further ultrasonication for 3 h. Afterward, the resulting mixture was centrifuged to collect the precipitate, which was subsequently dried under vacuum at 75 °C for 12 h.

### 2.4. Construction of the MB@SWCNHs/T-PEDOT/GCE

Prior to modification, the bare glassy carbon electrode (GCE) underwent sequential polishing with 0.1, 0.5, and 0.05 μm alumina (Al_2_O_3_) powder, followed by rinsing with ultrapure water. Subsequently, the electrode was immersed in a 5.0 mmol L^−1^ [Fe(CN)_6_]^4−/3−^ electrolyte solution containing 1.0 mol L^−1^ KCl, and cyclic voltammetry (CV) scanning was performed until the redox potential difference was less than 70 mV. After washing with ultrapure water, the electrode was dried and set aside. The fabrication process for the MB@SWCNHs/T-PEDOT/GCE was as follows: Initially, 2.0 mg of the prepared SWCNHs/T-PEDOT composites was uniformly dispersed in 2.0 mL of DMF solvent through ultrasonication. Subsequently, 40.0 μL of the previously prepared MB-DMF solution (1.0 mg mL^−1^) was added to the dispersion and sonicated for 15 min. Then, 7 μL of the resulting MB@SWCNHs/T-PEDOT mixture was applied dropwise to the surface of the bare GCE. After drying, 5 μL of 0.1% Nafion solution was further applied to the electrode surface to obtain the MB@SWCNHs/T-PEDOT/GCE.

### 2.5. Electrochemical Behavior of the MB@SWCNHs/T-PEDOT Sensing Interface

The electrochemical behavior of the developed MB@SWCNHs/T-PEDOT sensing interface was evaluated in a 5.0 mmol L^−1^ [Fe(CN)_6_]^4−/3−^ electrolyte solution containing 0.1 mol L^−1^ KCl using CV scanning, electrochemical impedance spectroscopy (EIS) analysis, and differential pulse voltammetry (DPV) scanning in a CHI440C and Princeton 2273 electrochemical workstation. CV measurements were performed at a scan rate of 20–300 mV s^−1^ within potential ranges of −0.2 to 0.6 V and −0.4 to 0.4 V. For EIS analysis, the frequency range was set from 0.01 to 10,000 Hz, with an amplitude of 5.0 mV.

### 2.6. Electrochemical Detection of TBHQ

The constructed MB@SWCNHs/T-PEDOT/GCE was employed for the electrochemical detection of the target TBHQ by the DPV method. A three-electrode system using the MB@SWCNHs/T-PEDOT/GCE as the working electrode, a Pt electrode as the counter electrode, and a saturated calomel electrode as the reference electrode was immersed in phosphate-buffered saline (PBS) solutions containing various concentrations of TBHQ at room temperature. DPV measurements were taken at a scanning voltage range of −0.4 to 0.4 V, a pulse width of 0.6 s, and a pulse amplitude of 50 mV.

### 2.7. Real Sample Analysis

In this study, wafer biscuits, peanut oil, and instant noodles were used as real samples to evaluate the capability of the MB@SWCNHs/T-PEDOT/GCE sensor to detect TBHQ in real samples. All samples tested were obtained from a local supermarket in Tianjin, China. Initially, 1.0 g of each sample was accurately weighed and placed in a 10.0 mL centrifuge tube, followed by the addition of 5.0 mL of ethanol. The resulting mixture was shaken vigorously for 5 min and then subjected to ultrasonication for 30 min. Subsequently, the supernatant was collected by centrifugation at 8000 rpm for 15 min. This extraction process was repeated twice, and the collected supernatants were combined, concentrated with nitrogen, and then made up to 10.0 mL with ethanol. Finally, after filtration through a 0.22 μm filter membrane, the amount of TBHQ was determined by electrochemical and HPLC methods.

The verification of TBHQ content using the HPLC method was in accordance with the GB/T 21512-2008 standard [24]. Chromatographic separation was performed on a C18 column (250 mm × 4.6 mm, 5 μm), with the mobile phase consisting of 5% acetic acid solution (A) and methanol/acetonitrile (1/1, *v*/*v*, B) mixture with a flow rate of 1.0 mL min^−1^. The elution gradient proceeded as follows: phase B gradually increased from 30% to 100% over min 0–8, was maintained at 100% from min 9 to 14, and then decreased from 100% to 30% over min 15–22. The column temperature, detection wavelength, and injection volume were set at 40 °C, 280 nm, and 20 μL, respectively.

## 3. Results and Discussion

### 3.1. Characterization of T-PEDOT, SWCNHs, and SWCNHs/T-PEDOT

**Figure 2:**
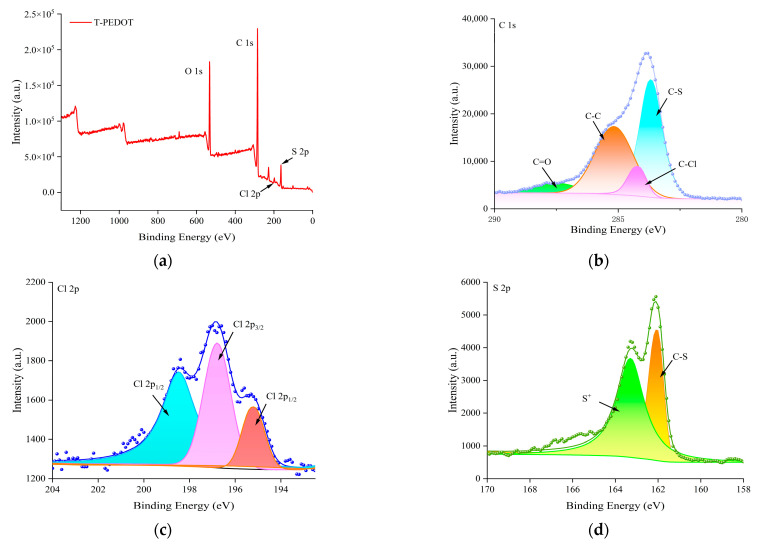
XPS spectra of T-PEDOT: (**a**) Survey scan; (**b**) C 1s; (**c**) Cl 2p; (**d**) S 2p.

**Figure 3:**
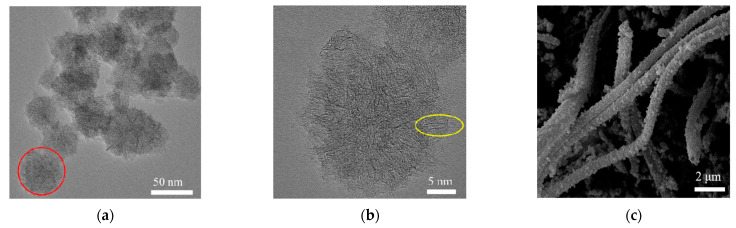

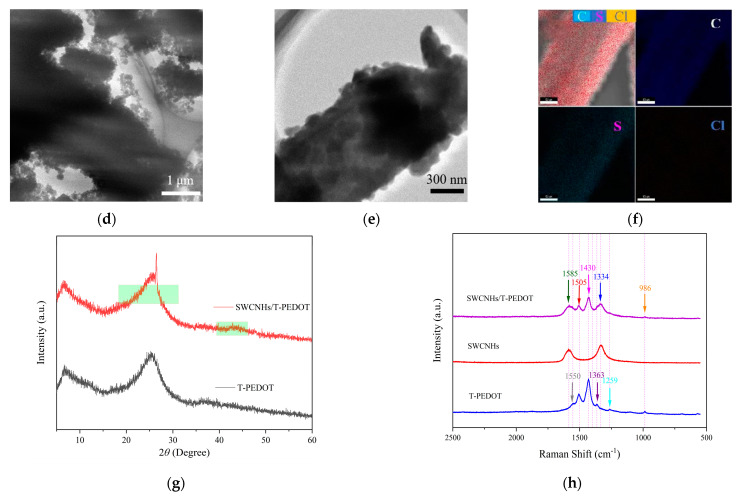
TEM of SWCNHs (**a**,**b**); SEM (**c**) and TEM (**d**,**e**) of SWCNHs/T-PEDOT under different magnifications. Mixed energy spectrum of SWCNHs/T-PEDOT and individual energy spectrum of C, S, and Cl (**f**); XRD of T-PEDOT and SWCNHs/T-PEDOT (**g**); Raman spectrum of T-PEDOT; SWCNHs and SWCNHs/T-PEDOT (**h**).

The elemental composition of the synthesized T-PEDOT was investigated using XPS analysis. Figure 2a confirms the presence of C, O, Cl, and S elements in the T-PEDOT material. The high-resolution spectra shown in Figure 2b of C 1s revealed distinctive peaks at 283.7 eV, 284.2 eV, 285.0 eV, and 287.7 eV corresponding to C-S, C-Cl, C-C, and C=O bonds, respectively. The Cl 2p spectra exhibited characteristic peaks at 195.2 eV and 198.5 eV, which corresponded to Cl 2p_1/2_ and Cl 2p_3/2_, respectively. The peak at 195.2 eV indicated the presence of Cl ions, while the peak at 198.5 eV suggested the presence of organic chlorine compounds, with 196.8 eV corresponding to the C-Cl bonds (Figure 2c). In the XPS spectrum of S 2p (Figure 2d), two distinct peaks were observed, corresponding to the C-S bonds (162.1 eV) and cation S^+^ (163.3 eV). The formations of C-S, C-Cl, C-C, and C=O bonds suggested that the T-PEDOT had been successfully synthesized. Figure 3a,b presented the TEM images of SWCNHs, illustrating the formation of “dahlia” cluster-like aggregate structures with diameters ranging from 30 to 60 nm. Due to the action of high-energy electron beams, the tube walls of SWCNHs were damaged, resulting in blurred edges [25]. Upon partial enlargement (Figure 3b), it was evident that the “petals” exhibited a tubular structure with a conical shape (yellow), while retaining a small minor graphite flake structure. The SEM image of SWCNHs/T-PEDOT in Figure 3c revealed a typical tubular structure with a rough surface. Subsequent TEM results (Figure 3d,e) further highlighted the presence of internal voids within the material and attributed the rough surface to the SWCNHs coating. The elemental mapping analysis (EDS) confirmed the distribution of primary elements C, S, and Cl within the SWCNHs/T-PEDOT material, providing conclusive evidence of successful synthesis (Figure 3f). The synthesized T-PEDOT and SWCNHs were further used to construct the ratiometric electrochemical sensor for TBHQ.

Figure 3g displays the XRD spectra of the synthesized T-PEDOT and SWCNHs/T-PEDOT materials. The T-PEDOT material exhibited prominent diffraction peaks at 6.59° and 25.14°, consistent with previous studies in the literature [26], indicating its successful synthesis and a disordered growth state. Meanwhile, the XRD patterns of the SWCNHs/T-PEDOT composite preserved the diffraction peak of T-PEDOT and featured a distinct sharp peak at 26.46°, which was attributed to graphene, confirming the presence of the typical graphene structure within SWCNHs as observed in the TEM images (Figure 3a,b). Furthermore, the SWCNHs/T-PEDOT material exhibited less prominent broad diffraction peaks at 26.00° and 42.76°, corresponding to the (002) and (100) crystal planes of the hexagonal graphite structure, respectively [27]. The results of Raman spectrum analysis of T-PEDOT, SWCNHs, and SWCNHs/T-PEDOT are presented in Figure 3h. The characteristic peaks observed at 986 cm^−1^ and 1259 cm^−1^ in the T-PEDOT Raman spectrum were attributed to the stretching vibration of the C-O-C bond and the vibration of the C-C bond in PEDOT. Additionally, the peaks at 1430 cm^−1^ and 1509 cm^−1^ corresponded to the symmetric stretching vibrations of the benzene-type and quinone-type C_α_=C_β_ bonds on the thiophene ring of PEDOT, respectively, while the peak at 1550 cm^−1^ corresponded to the antisymmetric stretching vibration of C_α_=C_β_. Compared to the benzene-type chain, the quinone-type PEDOT main chain promoted structural order in PEDOT molecules, providing enhanced carrier transfer and improved electrical conductivity [27]. The formed composite material retained the characteristic peaks of SWCNHs and T-PEDOT. Due to the strong interaction between the components, the characteristic peaks of the D band of SWCNHs at 1341 cm^−1^ and the G band at 1582 cm^−1^ shifted slightly to the right, appearing at 1343 cm^−1^ and 1585 cm^−1^, respectively [28]. The obvious differences in the XRD and Raman spectrum of the SWCNHs/T-PEDOT compared with T-PEDOT indicated that the materials have been successfully fabricated.

### 3.2. Electrochemical Behavior of the MB@SWCNHs/T-PEDOT/GCE

**Figure 4:**
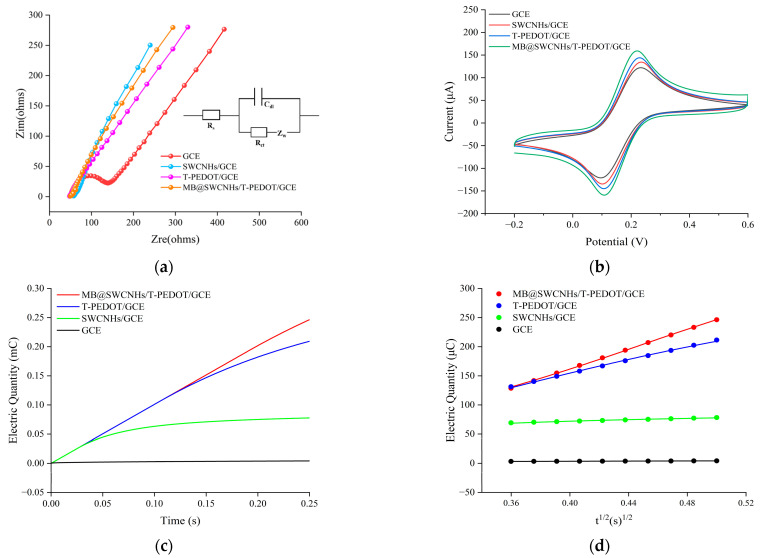
(**a**,**b**) EIS and CV of various modified electrodes in 5.0 mmol L^−1^ [Fe(CN)_6_]^4−/3−^ (containing 0.1 mol L^−1^ KCl); (**c**) Q-t curves of different modified electrodes in 0.1 mol L^−1^ KCl (containing 1.0 mmol L^−1^ [Fe(CN)_6_]^4−/3−^); (**d**) The relationship between Q and t^1/2^.

The electron transfer characteristics of various materials were investigated through EIS and CV scanning in a solution comprising 5.0 mmol L^−1^ [Fe(CN)_6_]^4−/3−^ and 0.1 mol L^−1^ KCl at a frequency of 0.01~10,000 Hz and an amplitude of 5.0 mV. In the Nyquist plot, the semicircle and linear segments, respectively, represent the electron transfer and diffusion processes occurring on the electrode surface, with the diameter of the semicircle correlating to the resistance value. The *R*_ct_ of the different electrodes were fitted by the equivalent circuit. Here, *R*_s_ stands for the solution resistance, *R*_ct_ represents charge transfer resistance, C_dl_ is the Warburg impedance, and *Z*_w_ refers to double-layer capacitance. As depicted in Figure 4a, the Nyquist curve of the bare GCE exhibited the largest semicircle diameter (*R*_ct_ = 80 Ω), indicating lower electron transfer ability and poor conductivity. Conversely, the modification of the electrode with materials such as T-PEDOT, SWCNHs, and SWCNHs/T-PEDOT resulted in a reduction in the semicircular portion of the Nyquist curve, indicating improved electron transfer ability at the electrode interface facilitated by the materials’ enhanced conductivity. The *R*_ct_ value of T-PEDOT/GCE was 30 Ω, while that of MB/SWCNHs/T-PEDOT/GCE was reduced to about 25 Ω. The impedance values of the SWCNHs/T-PEDOT-modified electrodes are much lower than those of the single material and the other materials (such as Ni-MOF/T-PEDOT [19] and SWCNHs@ZIF-67 [25]) mainly because of the synergistic catalytic effect of T-PEDOT and SWCNHs. The CV results (Figure 4b) revealed distinct quasi-reversible redox peak pairs in the [Fe(CN)_6_]^4−/3−^ electrolyte for each modified electrode. It is particularly worth mentioning that the oxidation peak current of the SWCNHs/T-PEDOT-modified electrode (168.9 μA) is higher than that of the SWCNHs- (147.9 μA) and T-PEDOT (158.6 μA)-modified electrodes. This observation indicated that the SWCNHs/T-PEDOT composite-modified electrode had a faster electron transfer rate, rendering it more suitable for electrochemical sensing applications. The chronocoulometric method was further employed to evaluate the effective working area of each modified electrode, as illustrated in Figure 4c,d, showcasing the corresponding linear relationship obtained for each electrode. According to Anson’s equation [28],

*Q* = (2 × *F* × *A* × *c* × *D*^1/2^*t*^1/2^)/*π*^1/2^ + *Q*_dl_ + *Q*_ads_

where *F* represents the Faraday’s constant (96,485 C mol^−1^), A denotes the effective working area, *c* represents the substrate concentration, and *D* represents the diffusion coefficient (1.0 mmol L^−1^ [Fe(CN)_6_]^4−/3−^: 7.6 × 10^−6^ cm^2^ s^−1^). *Q*_dl_ and *Q*_ads_ indicate the Faraday electric charge and the electric double-layer electric charge, respectively. The linear equations for various electrodes were as follows: GCE: *Q* (mC) = 0.007 *t*^1/2^ (*s*^1/2^) + 7.197, with *R*^2^ of 0.999; SWCNHs/GCE: *Q* (mC) = 0.064 *t*^1/2^ (*s*^1/2^) + 0.046, with R2 of 0.988; T-PEDOT/GCE: *Q* (mC) = 0.571 *t*^1/2^ (*s*^1/2^) − 0.074, with *R*^2^ of 0.998; MB@SWCNHs/T-PEDOT/GCE: *Q* (mC) = 0.839 *t*^1/2^ (*s*^1/2^) − 0.173, with *R*^2^ of 0.999. The effective working area was calculated as 0.0018 cm^2^ (GCE), 0.0021 cm^2^ (SWCNHs/GCE), 0.0191 cm^2^ (T-PEDOT/GCE), and 0.0281 cm^2^ (SWCNHs/T-PEDOT/GCE). These results indicated that the SWCNHs/T-PEDOT composite material significantly enhanced the effective working area of the electrode and provided more active sites, which facilitated achieving highly sensitive electrochemical detection of TBHQ.

### 3.3. Electrochemical Redox Mechanism of TBHQ

**Figure 5:**
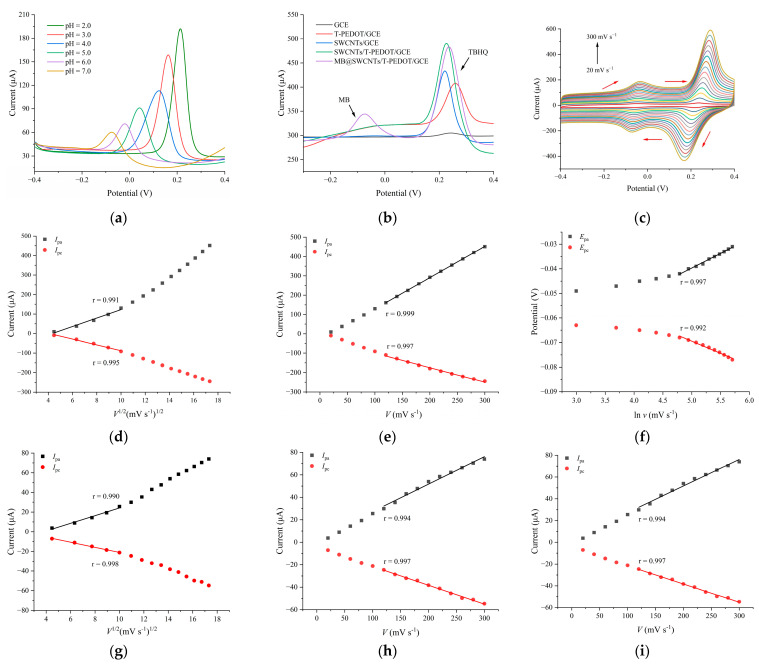
(**a**) DPVs of SWCNHs/T-PEDOT/GCE under various pH values (2.0–7.0); (**b**) DPV curves of different modified electrodes in 0.1 mol L^−1^ PBS (pH 2.0) containing 10.0 μg mL^−1^ TBHQ; (**c**) CVs of MB@SWCNHs/T-PEDOT/GCE in 0.1 mol L^−1^ PBS (pH 2.0) containing 10.0 μg mL^−1^ TBHQ at different scan rates (20–300 mV s^−1^). Plot of a linear relationship between TBHQ peak current (*I*_pa_, *I*_pc_) and scan rates (v^1/2^) (**d**) and scan rates (v) (**e**). Plot of a linear relationship between TBHQ peak potential (*E*_pa_, *E*_pc_) and the natural logarithm of the scanning rate (ln *v*) (**f**). Plot of a linear relationship between MB peak current (*I*_pa_, *I*_pc_) and scan rates (*v*^1/2^) (**g**) and scan rates (v) (**h**). Plot of a linear relationship between MB peak potential (*E*_pa_, *E*_pc_) and the natural logarithm of the scanning rate (ln *v*) (**i**).

The electrochemical oxidation mechanism of TBHQ, having part of the phenol group, typically involves proton transfer, leading to the generation of quinones. Therefore, the proton concentration in the buffer environment played a crucial role in influencing the electrochemical oxidation process. In this study, the DPV method was utilized to investigate the electrochemical response of the SWCNHs/T-PEDOT-modified electrode to TBHQ in PBS with varying pH. As illustrated in Figure 5a, the oxidation peak potential (*E*_pa_) of TBHQ exhibited a significant negative shift as the buffer pH increased from 2.0 to 7.0. Furthermore, the correlation between buffer pH and the *E*_pa_ of TBHQ revealed a robust linear relationship described by *E*_pa_ (V) = 0.345–0.060 pH (*R*^2^ = 0.989) (Figure S1). The slope (−60 mV pH^−1^) of this equation closely matched the theoretical value of −59.0 mV pH^−1^, suggesting that the TBHQ reaction at the electrode followed an isoelectron–isoproton transfer mechanism [29]. The DPV curves in Figure 5b for each modified electrode in PBS solution (0.1 mol L^−1^, pH 2.0) containing 10.0 μg mL^−1^ TBHQ provided important insights. The oxidation peak potential of TBHQ was found to be close to 0.22 V, with the bare GCE displaying a modest oxidation peak (*E*_pa_) and a peak current (*I*_pa_) of 6.94 μA. Upon modification of the GCE with T-PEDOT, possessing remarkable catalytic capability, the *I*_pa_ increased significantly to 84.35 μA, attributed to the conductivity and tubular features of T-PEDOT, which provided a larger surface area for improved target adsorption. The SWCNHs-modified GCE exhibited an *I*_pa_ for TBHQ of 183.50 μA, attributed to the catalytic capability and porosity of SWCNHs facilitating target diffusion to the electrochemical interface. The composite SWCNHs/T-PEDOT-modified electrode demonstrated the highest *I*_pa_ value for TBHQ at 202.80 μA, indicating a significant electrocatalytic efficiency of the composite material on TBHQ. This was attributed to the synergistic effect between T-PEDOT and SWCNHs: the high surface area of T-PEDOT served as an effective carrier for the loading of SWCNHs, preventing their aggregation on the sensing interface, while SWCNHs, with its surface defects, provided electroactive sites for TBHQ adsorption through π-π interaction. This facilitation of enhanced electron transfer during the TBHQ redox process significantly improved detection sensitivity. Furthermore, an oxidation peak at −0.076 V was observed for MB@SWCNHs/T-PEDOT/GCE, with the current remaining stable despite changes in the TBHQ concentration. The results indicate that incorporating MB does not interfere with the detection signal, establishing a foundation for a ratiometric electrochemical quantitative analyzer using MB as a reference for TBHQ detection.

Figure 5c displays the CV curves obtained for the fabricated MB@SWCNHs/T-PEDOT/GCE in PBS (0.1 mol L^−1^, pH 2.0) containing 10.0 μg mL^−1^ TBHQ at different scan rates (*v* ranging from 20 to 300 mV s^−1^). It is evident that with increasing scan rate (*v*), both the peak currents (*I*_pa_ and *I*_pc_) of TBHQ and MB increased accordingly. In addition, the peak potential values (*E*_pa_ and *E*_pc_) of TBHQ and MB shifted towards positive and negative potentials, respectively. At lower scan rates (20–100 mV s^−1^), the *I*_pa_ and *I*_pc_ values of TBHQ exhibited a linear correlation with the square root of the scan rate (*v*^1/2^) (Figure 5d,g). The linear equations derived from the relationship for TBHQ were *I*_pa_ (μA) = −93.781 + 21.647 *v*^1/2^ (mV s^−1^)^1/2^ (*r*^2^ = 0.991) and *I*_pc_ (μA) = 60.933–149.062 *v*^1/2^ (mV s^−1^)^1/2^ (*r*^2^ = 0.995), indicating that the electrode surface reaction followed a diffusion-controlled process at low scan rates. Conversely, at higher scan rates (100–300 mV s^−1^), the *I*_pa_ and *I*_pc_ of TBHQ exhibited a linear relationship with the scan rate v (mV s^−1^) (Figure 5e,h). The linear equations for TBHQ were *I*_pa_ (μA) = −33.140 + 1.619 *v* (mV s^−1^) (*r*^2^ = 0.999) and *I*_pc_ (μA) = −25.693–0.745 v (mV s^−1^) (*r*^2^ = 0.997), indicating that at higher scan rates, the electrochemical oxidation–reduction reaction of TBHQ on the MB@SWCNHs/T-PEDOT/GCE were adsorption-controlled processes. Figure 5f,i illustrate the correlation between *E*_pa_, *E*_pc_, and ln *v* of TBHQ. At higher scanning rates, the linear equations describing the relationship between *E*_pa_, *E*_pc_, and ln *v* were as follows: *E*_pa_ (V) = 0.095 + 0.0334 ln *v* (*r*^2^ = 0.996) and *E*_pc_ (V) = 0.348–0.031 ln *v* (*r*^2^ = 0.989), indicating the quasi-reversible electrochemical process of TBHQ on the MB@SWCNHs/T-PEDOT/GCE. According to the Laviron equation [30], the electron transfer coefficient α and the electron transfer number n of TBHQ were determined to be 0.48 and approximately 1.71 (rounded up to 2.0), respectively. This indicated that the electrochemical reaction of TBHQ on the MB@SWCNHs/T-PEDOT/GCE involves the transfer of approximately 2 electrons. The analysis confirmed that the overall surface reaction on the electrode involved an isoelectron–isoproton process, indicating that TBHQ underwent the transfer of two protons and two electrons during the electrochemical redox process on the MB@SWCNHs/T-PEDOT/GCE surface.

### 3.4. Experimental Parameter Optimizations

To improve the detection performance of the MB@SWCNHs/T-PEDOT/GCE for TBHQ, comprehensive optimization studies were carried out on a series of experimental parameters, including the ratio of T-PEDOT/SWCNHs, the amount of SWCNHs/T-PEDOT composite, the amount of 0.1% Nafion, the adsorption potential, time, and scan rate. Figure S2a shows the effects of different mass ratios of T-PEDOT and SWCNH on the TBHQ oxidation peak current (*I*_pa_). Notably, when the mass ratio of T-PEDOT/SWCNHs reached 1/2, there was a significant increase in the *I*_pa_ value compared to other ratios. Additionally, at a SWCNHs/T-PEDOT concentration of 1.0 mg mL^−1^, increasing the dispensing volume from 3 μL to 7 μL resulted in a gradual increase in the *I*_pa_ of TBHQ. However, after exceeding 7 μL, the *I*_pa_ started to decrease due to the excessive thickness of the modified material’s film layer, which hindered electron transfer at the electrode interface (Figure S2b). To ensure the stability of the constructed sensor, a 0.1% Nafion solution was used to anchor the modified materials onto the electrode surface. As shown in Figure S3, the *I*_pa (TBHQ)_/*I*_pa (MB)_ ratio consistently decreased as the dispensing volume of 0.1% Nafion (5–10 μL) increased. A dispensing volume of 5 μL of Nafion (0.1%) effectively stabilized the material on the electrode surface and was consequently selected as the optimal condition. The effect of various adsorption potentials (−0.15–0.10 V) and durations (25–325 s) on the *I*_pa (TBHQ)_/*I*_pa (MB)_ ratio was investigated, and the results are presented in Figure S4a,b. It was observed that when the same conditions were maintained, the *I*_pa (TBHQ)_/*I*_pa (MB)_ ratio reached its peak at an adsorption potential of 0.0 V, indicating the optimal adsorption potential. Additionally, the *I*_pa (TBHQ)_/*I*_pa (MB)_ ratio gradually increased with extended adsorption time, stabilizing after 180 s, indicating the optimal duration of the process.

Figure S5 shows the DPV response curves of the prepared MB@SWCNHs/T-PEDOT/GCE when exposed to the target TBHQ in different buffers, including Tris buffer, acetate buffer (NaAc-HAc), Britton–Robinson (BR) buffer and PBS (pH 2.0). The results demonstrate that clear and pronounced oxidation peaks for TBHQ were observed in PBS and NaAc-HAc, with the peak current for TBHQ oxidation reaching its maximum in PBS (183.7 μA). In contrast, oxidation peaks for TBHQ were virtually absent in the BR and Tris-HCl electrolytes.

### 3.5. Analytical Performance of the Ratiometric Electrochemical Sensing Platform

**Figure 6:**
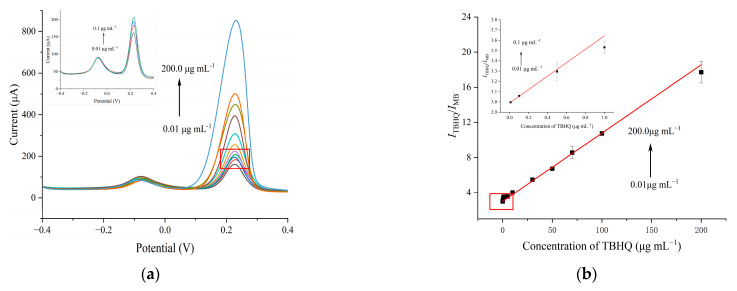

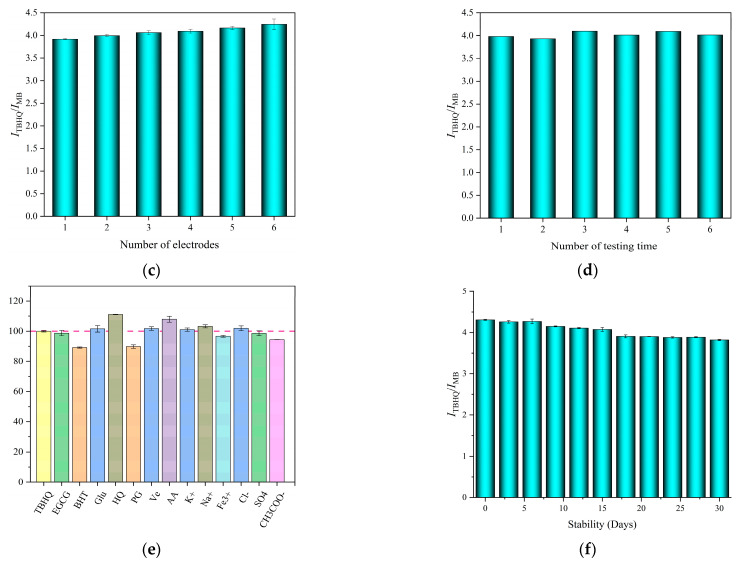
(**a**) DPV curves of MB@SWCNHs/T-PEDOT/GCE at different concentrations of TBHQ (0.01–200.0 μg mL^−1^). Inset: enlarged DPV curves from 0.01 to 0.1 μg mL^−1^. (**b**) Plot of the ratio of the peak current of TBHQ and MB (*I*_TBHQ_/*I*_MB_) vs. the concentration of TBHQ (0.1–200.0 μg mL^−1^). Evaluation of MB@SWCNHs/T-PEDOT/GCE: (**c**) reproducibility; (**d**) repeatability; (**e**) anti-interference ability, and (**f**) Stability.

Under the optimized experimental conditions, the analytical capabilities of the developed electrochemical sensing strategy for TBHQ were evaluated using the DPV method. Figure 6a displays the oxidation peaks of MB and TBHQ at −0.076 V and 0.228 V, respectively. It is observed that the oxidation peak currents of TBHQ increased with increasing concentrations of TBHQ, whereas the oxidation peak current of MB remained largely unchanged. Figure 6b shows that the ratio of TBHQ and MB oxidation peak currents (*I*_TBHQ_/*I*_MB_) is directly proportional to the concentration of TBHQ (*C*_TBHQ_). The relationship is quantified by the following regression equations:

(*C*_TBHQ_: 0.01–0.1 μg mL^−1^): *I*_TBHQ_/*I*_MB_ (μA) = 0.653 *C*_TBHQ_ (μg mL^−1^) + 2.987 (*R*^2^ = 0.999)

(*C*_TBHQ_: 0.1–200.0 μg mL^−1^): *I*_TBHQ_/*I*_MB_ (μA) = 0.074 *C*_TBHQ_ (μg mL^−1^) + 3.320 (*R*^2^ = 0.998)

Figure 6a,b demonstrate that the sensitivity of the sensor for detecting TBHQ was enhanced at low concentrations (0.01–0.1 μg mL^−1^). This might be attributed to the predominance of adsorption-controlled characteristics at the electrode interface at lower TBHQ concentrations. In contrast to other analytes, the abundant catalytic sites on the electrode surface facilitate the rapid catalytic oxidation of TBHQ to *tert*-butylhydroquinone (TQ). In the high concentration range (0.1–200.0 μg mL^−1^), sensitivity to TBHQ declined. This reduction was due to a considerable amount of TBHQ accumulating on the electrode surface, leading to an increase in mass transfer resistance and a decrease in available catalytic sites. In comparison with previously reported electrochemical sensing strategies for TBHQ (Table 1), the proposed ratiometric electrochemical sensor MB@SWCNHs/T-PEDOT/GCE possessed a broader linear range (0.01–200.0 μg mL^−1^) and a lower detection limit (LOD, 0.005 μg mL^−1^). The wide detection range and high sensitivity of our reported method for TBHQ shows great promise for application in food safety and environment analysis. The excellent analytical performance for TBHQ is mainly attributed to the high conductivity and catalytic capacity of T-PEDOT.

To precisely assess the reproducibility of the ratiometric electrochemical sensing platform, a series of MB@SWCNHs/T-PEDOT/GCE-modified electrodes were meticulously fabricated and employed for TBHQ detection at a constant concentration of 10.0 μg mL^−1^. The DPV results (*I*_TBHQ_/*I*_MB_) were determined, as depicted in Figure 6c. The relative standard deviation (RSD) of the six parallel measurements was 2.9% (n = 3), suggesting that the constructed sensor possesses remarkable repeatability. Additionally, the identical MB@SWCNHs/T-PEDOT/GCE was utilized to concurrently determine TBHQ (10.0 μg mL^−1^) six times, and the calculated RSD was 1.7% (n = 6), indicating good reproducibility in TBHQ quantification (Figure 6d).

During the TBHQ detection process, interference from the food matrix will affect the redox signal. To evaluate the anti-interference ability of the method, the DPV current of this developed MB@SWCNHs/T-PEDOT/GCE sensor was examined in the presence of various concentrations of structural analogs, antioxidants (10.0 μg mL^−1^), and common metal and acid radical ions (50.0 μg mL^−1^), including EGCG, BHT, Glu, HQ, PG, VE, AA, K^+^, Na^+^, Fe^3+^, Cl^−^, SO_4_^2−^, and CH_2_COO^−^. As shown in Figure 6e, in the presence of interferents, the ratiometric response to TBHQ remained within 90–110% of the original value. This indicated that the constructed ratiometric electrochemical sensing platform had good anti-interference capability for the detection of TBHQ. After storage at room temperature for 30 days, the DPV current response of the designed ratiometric electrochemical sensor with the same concentration of TBHQ remained at 88.7% (Figure 6f). These results underscore the superior reproducibility, repeatability, and stability of the proposed MB@SWCNHs/T-PEDOT/GCE sensor, demonstrating the feasibility of quantitative detection of TBHQ.

### 3.6. Real Sample Detection

Wafer biscuits, peanut oil, and instant noodles were deliberately spiked with three different levels of TBHQ (0, 10, 50 μg mL^−1^) and analyzed to verify the applicability of the proposed ratiometric electrochemical sensing platform. As presented in Table 2, the recoveries of TBHQ in the three selected samples ranged from 92.2 to 103.0%, accompanied by low RSDs (≤4.1%, n = 3). This outcome indicated that the constructed ratiometric sensor had good accuracy and reliability for the quantitative analysis of TBHQ in complex food matrices.

## 4. Conclusions

In this study, the T-PEDOT composites integrated with SWCNHs were employed to fabricate a rapid and highly sensitive ratiometric electrochemical sensing device specifically tailored for antioxidant TBHQ detection in food. The proposed SWCNHs/T-PEDOT-modified GCE displayed good electrocatalytic properties for the target TBHQ, which was mainly attributed to the remarkable synergistic effects between SWCNHs and T-PEDOT with electrical conductivity and specific surface area. The constructed ratiometric electrochemical sensor achieved high sensitivity and reliability in the detection of TBHQ. After appropriate modification, this ratiometric electrochemical sensing strategy could be applied to detect other targets with conductive properties, showing great potential for food safety and environmental analyses.

## Figures and Tables

**Table 1:** Comparisons of the different electrochemical strategies for TBHQ determination.

Modified Electrode	Method	Linear Range (μg mL^−1^)	LOD (μg mL^−1^)	Refs
MIP/PdAuNPs/ERGO/GCE ^1^	DPV	0.5–60	0.046	[31]
Co_3_O_4_@PPy/GCE ^2^	DPV	0.03–100	0.008	[32]
ZnCuMg TMO/β-CD-CB/SPCE ^3^	DPV	2.0–20.0	0.0001	[33]
MIP/ZC/GCE ^4^	DPV	0.02–12.5	0.07	[34]
MB@SWCNHs/T-PEDOT/GCE	DPV	0.01–0.1 0.1–200.0	0.005	This work

^1^ MIP/PdAuNPs/ERGO/GCE: molecularly imprinted/PdAu bimetal/electrochemical reduction graphene oxide-modified electrode. ^2^ Co_3_O_4_@PPy/GCEb: core-shell Co_3_O_4_@PPy-modified electrode. ^3^ ZnCuMg TMO/β-CD-CB/GCE: zinc–copper–magnesium ternary metal oxide/β-cyclodextrin-functionalized carbon black-modified electrode. ^4^ MIP/ZC/GCE: molecularly imprinted/ZIF-8-derived porous carbon-modified electrode.

**Table 2:** Results of TBHQ in real samples measured by MB@SWCNHs/T-PEDOT/GCE.

Samples	Spiked (μg mL^−1^)	Found (μg mL^−1^)	Recovery (%)	RSD (%, n = 3)
Wafer biscuits	0	0.29	-	4.1
	10	10.06	103.0	1.5
	50	49.63	98.7	1.0
Peanut oil	0	Not detected	-	-
	10	9.29	92.3	1.3
	50	46.11	92.2	2.5
Instant noodles	0	0.85	-	2.9
	10	10.28	102.8	1.8
	50	50.77	101.5	0.8

## Data Availability

The original contributions presented in the study are included in the article and Supplementary Materials, further inquiries can be directed to the corresponding author.

## Supplementary Materials

The following supporting information can be downloaded at: https://www.mdpi.com/article/10.3390/foods13182996/s1, Figure S1: The relationship between oxidation peak potential and different pH; Figure S2: Influence of (a) m(T-PEDOT)/m(SWCNHs) and (b) drop amounts of SWCNHs/T-PEDOT; Figure S3: Drop amounts of 0.1% Nafion; Figure S4: Accumulation potential and time; Figure S5: Effects of different buffers on oxidation peak currents of TBHQ.
