# Supplementary material for: Ratiometric Electrochemical Sensor Applying SWCNHs/T-PEDOT Nanocomposites for Efficient Quantification of Tert-Butylhydroquinone in Foodstuffs
Source: Foods. 2024 Sep 21;13(18):2996. doi: 10.3390/foods13182996 (PMC11431831; doi:10.3390/foods13182996)
Supplement: Supplementary file 1 [file foods-13-02996-s001.zip › foods-3212011-supplementary.pdf]

Supporting Materials

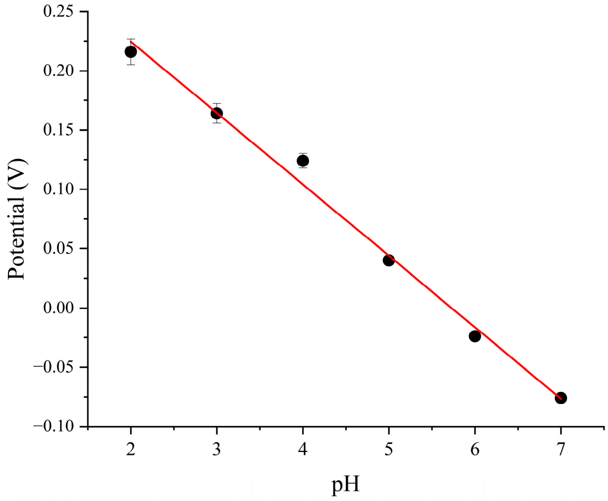

Figure S1. The relationship between oxidation peak potential and different pH.

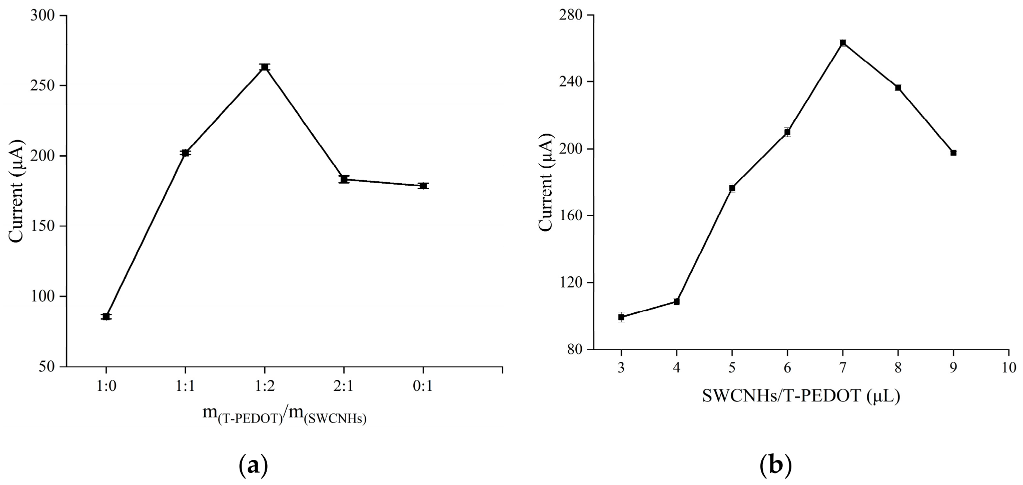

Figure S2. Influence of (a) $m_{(T\text{-}PEDOT)}/m_{(SWCNHs)}$; (b) Drops amount of SWCNHs/T-PEDOT (1.0 mg mL^{-1}).

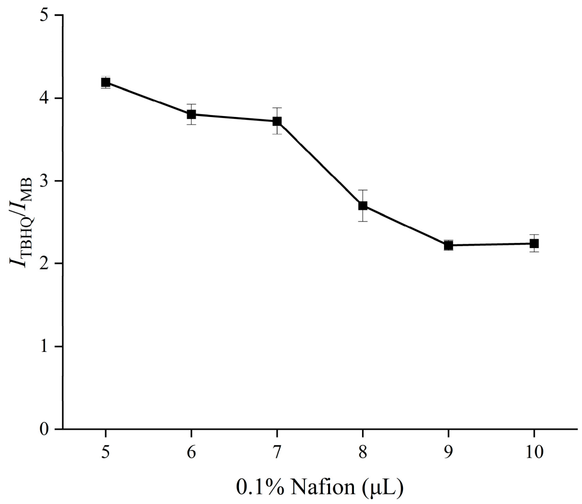

Figure S3. Drops amount of 0.1% Nafion on I_{TBHQ}/I_{MB}.

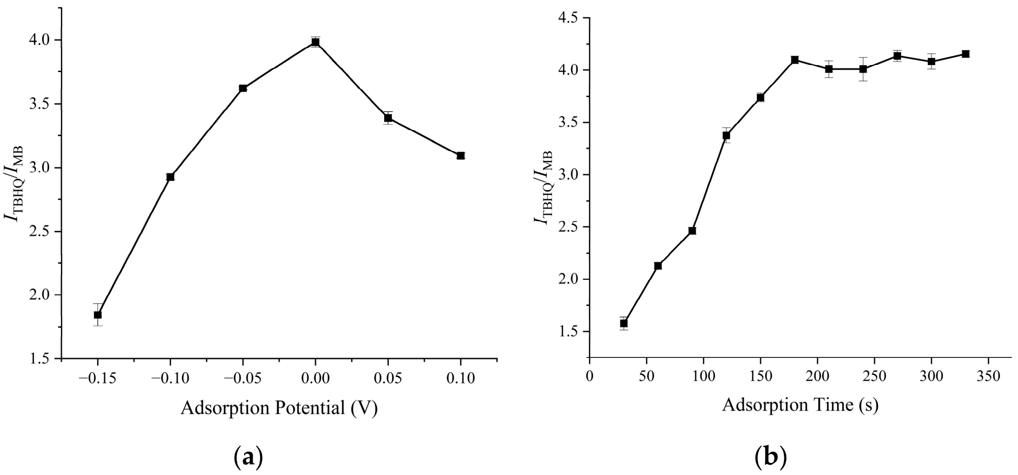

Figure S4. (a) Accumulation potential; (b) Accumulation time.

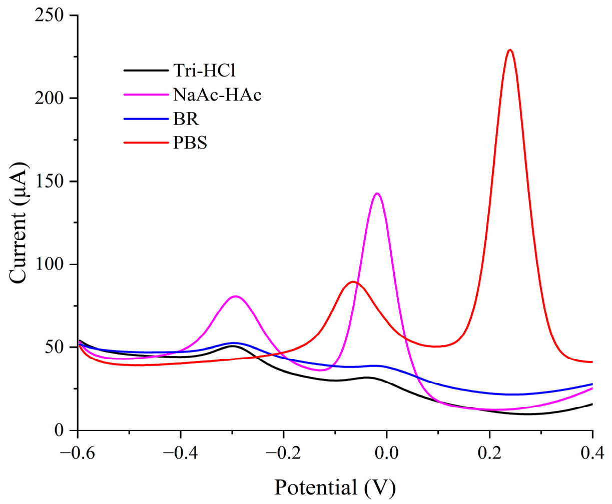

Figure S5. Different buffers on oxidation peak currents of TBHQ.
